# 5-HT_4_ Receptor Agonist Effects on Functional Connectivity in the Human Brain: Implications for Procognitive Action

**DOI:** 10.1016/j.bpsc.2023.03.014

**Published:** 2023-11

**Authors:** Angharad N. de Cates, Marieke A.G. Martens, Lucy C. Wright, Daisy Gibson, Gershon Spitz, Cassandra D. Gould van Praag, Sana Suri, Philip J. Cowen, Susannah E. Murphy, Catherine J. Harmer

**Affiliations:** a Department of Psychiatry, University of Oxford, Warneford Hospital, Oxford, United Kingdom; b Oxford Health NHS Foundation Trust, Warneford Hospital, Oxford, United Kingdom; c Monash-Epworth Rehabilitation Research Centre, Turner Institute for Brain and Mental Health, School of Psychological Sciences, Monash University, Clayton, Victoria, Australia; d Department of Neuroscience, Central Clinical School, Faculty of Medicine, Nursing and Health Sciences, Monash University, Clayton, Victoria, Australia; e Oxford Centre for Human Brain Activity and Oxford Centre for Functional MRI of the Brain, Wellcome Centre for Integrative Neuroimaging, Department of Psychiatry, University of Oxford, Oxford, United Kingdom

**Keywords:** Cognition, fMRI, Procognitive, Prucalopride, Serotonin, 5-HT_4_

## Abstract

**Background:**

Cognitive deficits are often comorbid with mood disorders and can cause significant functional impairment even after resolution of the primary mood symptoms. We do not currently have pharmacological treatments that adequately address these deficits. 5-HT_4_ receptor agonists show promise as potential procognitive agents in animal and early human translational studies. Optimal cognitive performance in humans is directly associated with appropriate functional connectivity between specific resting-state neural networks. However, so far the effect of 5-HT_4_ receptor agonism on resting-state functional connectivity (rsFC) in the brain in humans is unknown.

**Methods:**

We collected resting-state functional magnetic resonance imaging scans from 50 healthy volunteers, of whom 25 received 6 days × 1 mg prucalopride (a highly selective 5-HT_4_ receptor agonist) and 25 received placebo in a randomized double-blind design.

**Results:**

Network analyses identified that participants in the prucalopride group had enhanced rsFC between the central executive network and the posterior/anterior cingulate cortex. Seed analyses also showed greater rsFC between the left and right rostral anterior cingulate cortex and the left lateral occipital cortex, and reduced rsFC between the hippocampus and other default mode network regions.

**Conclusions:**

Similar to other potentially procognitive medications, low-dose prucalopride in healthy volunteers appeared to enhance rsFC between regions involved in cognitive networks and reduce rsFC within the default mode network. This suggests a mechanism for the behavioral cognitive enhancement previously seen with 5-HT_4_ receptor agonists in humans and supports the potential for 5-HT_4_ receptor agonists to be used in clinical psychiatric populations.

SEE COMMENTARY ON PAGE 1068

Around 1 in 5 people in their lifetime will develop major depressive disorder with significant morbidity and mortality (1). Cognitive deficits as part of depression are common and disabling, present in at least two-thirds or greater of those with a diagnosis (2,3), and are rated by patients as their greatest concern after low mood (4,5). A range of cognitive deficits are typically present in depression, such as poor memory, difficulties with attention and concentration, impaired executive functioning, reduced processing speed, and reduced ability to learn new information (6, 7, 8, 9). As well as reducing quality of life, cognitive problems also increase the risk of impaired social functioning and unemployment (10) and are economically costly (11,12).

Despite this clear unmet need, first-line therapeutic agents do not always successfully target impaired cognition specifically as part of mental illnesses such as mood or psychotic disorders (13,14). For example, in a recent study, 12 weeks of escitalopram (a selective serotonin reuptake inhibitor) was associated with a general improvement in cognition in patients with depression, but this was independent of any change in mood (15). Therefore, as cognitive impairments appear to be separable mechanistically from the primary symptoms of depressed mood and anhedonia, this may explain why up to 50% of people whose depression has otherwise remitted continue to experience day-to-day cognitive problems (6). The antidepressant drug vortioxetine is licensed in the United States specifically for cognitive impairment in depression due to the evidential promise of early studies (16, 17, 18, 19), details of which are present in the European summary of product characteristics updated in 2015 (20), although more recent data have not consistently replicated statistical superiority in comparison with selective serotonin reuptake inhibitors (21,22).

5-HT_4_ G protein–coupled receptors are found postsynaptically in brain regions including the frontal cortex, basal ganglia, and hippocampus (23,24). They also indirectly control the firing rate of serotonergic midbrain cells (25) and affect neuronal function by rapidly releasing neuroplasticity-related proteins (such as brain-derived neurotrophic factor) (26,27) and modulating the release of other neurotransmitters [including GABA (gamma-aminobutyric acid) (28), dopamine (29), and acetylcholine (30, 31, 32)]. As 5-HT_4_ receptor agonists can induce both brain-derived neurotrophic factor and acetylcholine release, they have the potential to ameliorate suggested neural and neurochemical abnormalities implicated in cognitive impairment in depression.

Preclinical data suggest that stimulating 5-HT_4_ receptors rapidly improves learning and memory in rodent models (33). This finding has been demonstrated using different 5-HT_4_ receptor agonists, and across a range of cognitive paradigms (26,34, 35, 36, 37), and effects are maintained for up to 14 to 30 days (38,39). We have also previously translated this effect to healthy humans: A single dose of the highly selective 5-HT_4_ receptor agonist prucalopride increased cognitive function across 3 different learning and memory tasks (the auditory verbal learning task, probabilistic instrumental learning task, and emotional memory within the Emotional Test Battery) (40). After repeated dosing for 6 days, using the same participants as the current study, prucalopride increased neural activation in memory-associated regions (the hippocampus and right angular gyrus) during memory tasks with simultaneous improvement in hippocampal-dependent memory task performance (41).

People with current and previous symptoms of depression also show functional changes within resting-state neural networks related to cognition compared with healthy volunteers, including the default mode network (DMN) (or medial frontoparietal network), central executive network (CEN) (or lateral frontoparietal network), and salience network (SN) (or midcinguloinsular network) (42, 43, 44). These appear to occur in both directions depending on the context (i.e., decreased resting-state functional connectivity [rsFC] between the DMN and CEN and increased rsFC between the SN and the CEN) (44), and thus these functional networks and their interactions are known as the triple network model (45). Large human datasets also show that appropriate connections between these specific networks are directly associated with successful cognitive performance (46).

In healthy volunteers, procognitive medication appears to lead to both increased and decreased connectivity including generally reduced rsFC between regions of the DMN (47) and increased rsFC in regions involved in cognition outside of the DMN, particularly in parts of the prefrontal cortex (48,49). Consistent with this, we found that 6 days of prucalopride enhanced inhibition of areas within the DMN (50) and led to increased activity in memory processing regions (41) while performing directed cognitive tasks with improved accuracy. The regions that we have found previously modulated by prucalopride in the context of cognitive task performance include the anterior cingulate cortex (ACC), posterior cingulate cortex (PCC), hippocampus, and angular gyrus.

Therefore, we wished to explore if subacute (6 days) prucalopride in healthy humans would influence rsFC of regions involved in cognitive processing (the triple network, and seeds involving the ACC, PCC, hippocampus, and angular gyrus). We did this using additional data from the same study reported in de Cates *et al.* (41,50). Based on existing evidence and our previous results (41,50), we hypothesized that prucalopride would reduce rsFC of the DMN and may have effects on other cognitive networks. Due to its size, functional heterogeneity (the ACC is thought to be linked with both emotional networks and cognitive networks), and the importance in our whole-brain and previous analyses of data within this study (50), a priori, we divided the ACC into 2 structural regions for seed placement: 1) a rostral portion involved with prefrontal regions and affective networks (including the DMN) and 2) and a caudal portion linked with sensorimotor regions and frontoparietal networks (51,52).

## Methods and Materials

### Participants

Healthy right-handed volunteers on no psychotropic medication (*N* *=* 50, age 18–40 years) were randomized to placebo or prucalopride (7 days × 1 mg [with imaging occurring on day 6]). Study participants and inclusion/exclusion criteria have been described in detail previously (41,50). The study protocol was preregistered with ClinicalTrials.gov (NCT03572790) and received approval from the University of Oxford Central University Research Ethics Committee (MSD-IDREC Reference No. R57219/RE001). No changes to methods occurred after the start of the study, and the flow of participants is outlined in Figure S1.

### Design and Randomization

The study had a between-subjects, double-blind, placebo-controlled design (41,50). Participants were randomly assigned 1:1 to prucalopride (Resolor) or placebo (lactose tablets; Rayonex Medical) for 7 days. Randomization was stratified for sex, with a block size of 4 (sealedenvelope.com; randomization code created June 1, 2018). Participants, investigators, and assessors were not aware of group allocation, and prucalopride and placebo capsules appeared identical.

Participants had a 3T scan, which included functional magnetic resonance imaging (fMRI) tasks on day 6, as participants were then at steady state in terms of prucalopride (53). The complete scanning protocol, including acquisition parameters and radiographer’s protocol is available online (see https://doi.org/10.5281/zenodo.6107725). In brief, this included a structural T1-weighted scan, 2 fMRI tasks [a faces emotion recognition and a memory-encoding task, results reported in (41,50)], a pseudocontinuous arterial spin labelling scan [results reported in (41)], and a resting-state blood oxygenation level–dependent fMRI scan. Female participants were not tested during their premenstrual week. Resting-state data acquisition was performed with participants being asked to relax but keep their eyes open. Data in this article relate to prespecified secondary outcomes.

### Questionnaire Measures and Behavioral Analysis

Mood, anxiety, personality, and side effects were examined with self-report questionnaires at baseline (screening), preimaging, and postimaging (day 6). Participants were asked to guess group allocation at the end of the study. Analysis of questionnaire data was previously reported (41).

### MRI Data

#### Acquisition

Blood oxygenation level–dependent fMRI and T1-weighted anatomical images were acquired using a 3T Siemens Prisma scanner (Siemens Corp.) with a 32-channel head matrix coil, as described previously (41,50). Here, we report results from the resting-state sequence of the imaging protocol. A total of 220 volumes of multiecho multiband (multiband factor = 3) resting-state fMRI were acquired with a voxel resolution of 2.5 × 2.5 × 2.5 mm^3^; −30 angulation; repetition time of 1600 ms; echo times of 15 ms (echo 1), 36 ms (echo 2), and 57 ms (echo 3); flip angle of 70°; and parallel imaging (parallel acquisition technique mode) of generalized autocalibrating partially parallel acquisitions 2. Scan duration was 6 minutes, 5 seconds. T1-weighted images were acquired with a voxel resolution of 1 × 1 × 1 mm^3^, and repetition time and echo time were 1900 ms and 3.97 ms, respectively. Gradient echo phase and magnitude field maps were acquired with voxel resolution of 2.5 × 2.5 × 2.5 mm^3^ to allow for distortion correction. Further details can be found in the Supplement and the full published scanning protocol.

#### Resting-State fMRI Analysis

Details of preprocessing are given in the Supplement.

#### Network Analysis

The preprocessed cleaned functional data were temporally concatenated across subjects and decomposed into independent components using FSL MELODIC (Multivariate Exploratory Linear Optimized Decomposition into Independent Components) (54). Dimensionality estimation for group maps was set to 20 independent component maps. They were identified as either being analogous to the most frequently reported major resting-state networks (RSNs) (55) or reflecting noise (physiological, scanner, movement). The components were first visually inspected, independently by ANdC and MAGM and subsequently by consultation with SS as appropriate, and then additionally inspected using Pearson spatial cross-correlation to compare quantitatively with previously published maps (see Table S1) (55). Dual regression was used to generate subject-specific versions of spatial maps and the associated time series from group-average spatial maps (54,56). Subsequently, we tested for statistically significant differences between the groups across all identified networks using FSL’s randomize permutation testing tool (5000 permutations). The RSNs of interest for this analysis were from the triple network model, comprising the DMN, SN, and CEN. Other RSNs (i.e., visual, auditory) were analyzed as control regions where we did not expect to see changes with prucalopride. Statistics were assessed using threshold-free cluster enhancement approach and a familywise error–corrected cluster significance threshold of *p <*  .05 applied to the suprathreshold clusters to correct for multiple comparisons at the voxel level (57). The general linear model included the groups of interest for comparison: placebo > prucalopride and prucalopride > placebo. To further visualize the results, individual parameter estimate values were extracted from their custom maps, using significant clusters as binary masks.

#### Seed Analysis

**Figure 1:**
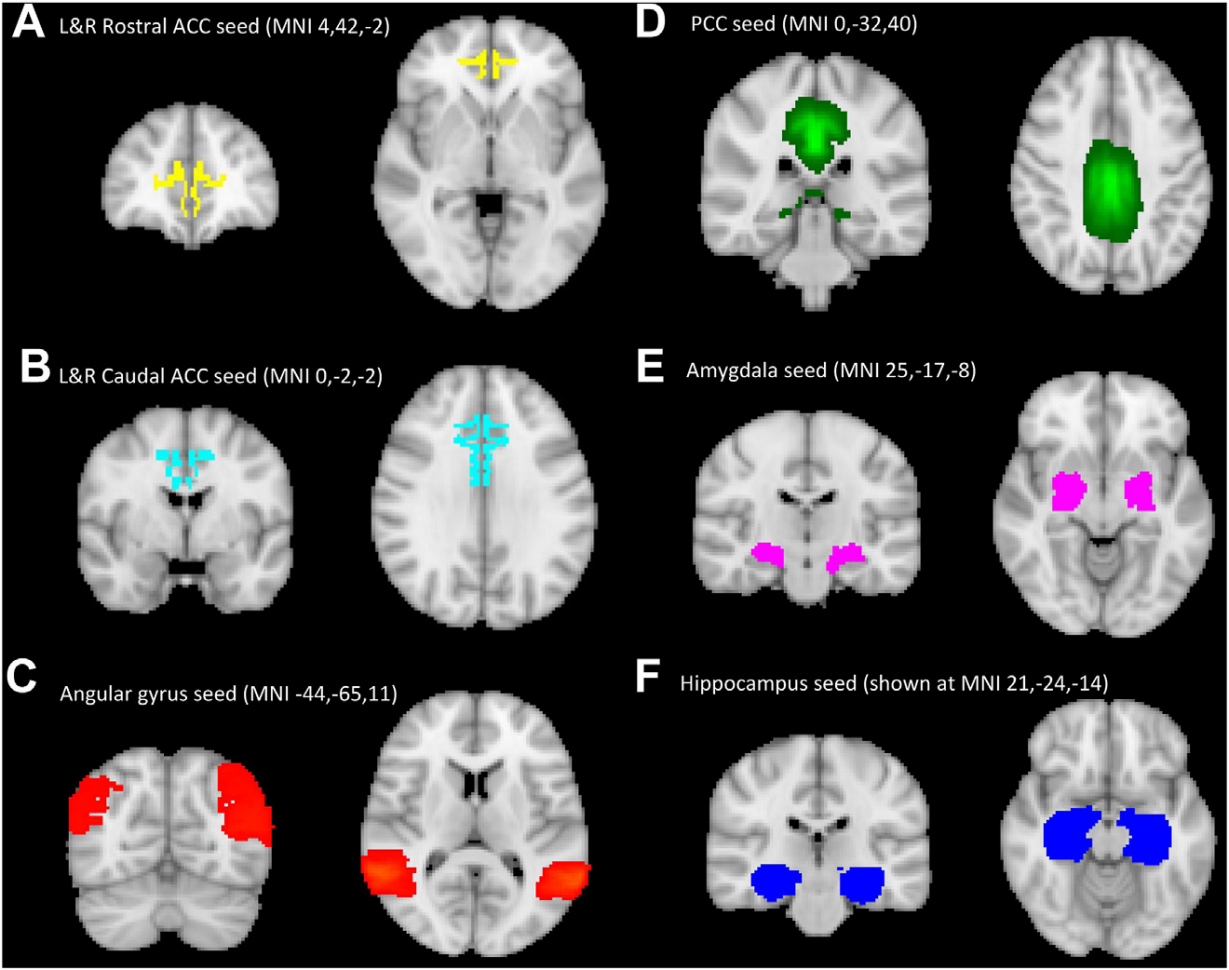
Seed maps used for seed analysis. Seed maps are shown in axial, coronal, and sagittal views. Left- and right-sided seeds shown together. ACC, anterior cingulate cortex; L, left; MNI, Montreal Neurological Institute; PCC, posterior cingulate cortex; R, right.

For the seed analysis, predetermined region-specific masks were chosen based on the previous literature: the ACC, PCC, hippocampus, amygdala, and angular gyrus. Masks were based on the FSL Harvard-Oxford atlas (https://fsl.fmrib.ox.ac.uk/fsl/fslwiki/FSL). As detailed above, the ACC was subdivided into different seeds along functional lines as per the FSL Talairach atlas: a rostral affective ACC (Brodmann areas: rostral 24, 25; rostral 32, 33) and a caudal cognitive ACC or midcingulate cortex (Brodmann areas: caudal 24, caudal 32) on both the left and the right (see Figure 1) (51,52,58). The Talairach atlas was used due to its increased specificity for this purpose. Masks were binarized and thresholded at 50% before creating a standard- to high-resolution matrix, which was applied to each mask for each participant in turn to register the mask into each individual’s functional (echo-planar imaging) space. We then extracted the time series for each mask for each participant. First-level connectivity was calculated as the correlation (both positive and negative) of time series of the seed with all other voxels in the brain using FSL FEAT (https://fsl.fmrib.ox.ac.uk/fsl/fslwiki/FEAT). A white matter and cerebral spinal fluid (CSF) mask were created in standard space and then registered into the individual’s functional (echo-planar imaging) space before being included as covariates of no interest. We then used FSL FEAT to perform group-level analysis with 2 explanatory variables (prucalopride vs. placebo) testing for the contrasts placebo > prucalopride and prucalopride > placebo. Cluster-based thresholding (*z* > 3.1, familywise error *p <* .05) was used to identify significant clusters for each seed analysis. To further visualize results, individual parameter estimate values were extracted from their custom maps, using significant clusters as binary masks. All results are reported using Montreal Neurological Institute (MNI) coordinates, and Bonferroni correction was applied to correct for multiple comparisons considering the 6 seeds used for analysis.

Distortion- and motion-corrected resting perfusion maps in units of mL/100 g/min were calculated using Oxford_ASL (part of the BASIL [Bayesian Inference for Arterial Spin Labelling] tool; https://fsl.fmrib.ox.ac.uk/fsl/fslwiki/BASIL) for each participant. Gray matter maps were also generated (using feat_gm_prepare) and included as a voxelwise covariate of no interest. Full details of the methods are reported in de Cates *et al.* (41) and the Supplement.

## Results

### Participants

All 50 (100% of target) participants were recruited between June 11, 2018, and May 17, 2019. As reported in de Cates *et al.* (41), 1 participant randomized to placebo was excluded from all analyses for data quality concerns noted at data collection. Two other participants randomized to prucalopride were excluded from fMRI analyses during the scan for acute anxiety and persistent sleepiness, although preexisting factors may have contributed to these outcomes. Another 3 participants were excluded from analyses for 1) a structural brain variant affecting registration to standard space, 2) MRI Quality Control tool assessment indicating a poor-quality structural scan (59), and 3) significant motion during the scan.

The final sample consisted of 44 participants (21 placebo, 23 prucalopride; age 18–36 years) [see (41) for full report of the group-level descriptives]. By chance, non-native English speakers were more likely to be in the prucalopride group. Participants appeared to be better than chance at guessing group allocation, particularly in the placebo group (correct guess: placebo 75.0%, prucalopride 60.9% [data missing for 1 participant]). No adverse events were recorded in the prucalopride group; 1 person in the placebo group discontinued the study due to abdominal discomfort. As previously reported in de Cates *et al.* (41), baseline and follow-up (day 6 pre- and postscan) mood, anxiety, and side effects showed no difference between the prucalopride and placebo groups (all *p*s > .5).

### Resting-State fMRI

#### Network Analysis

**Figure 2:**
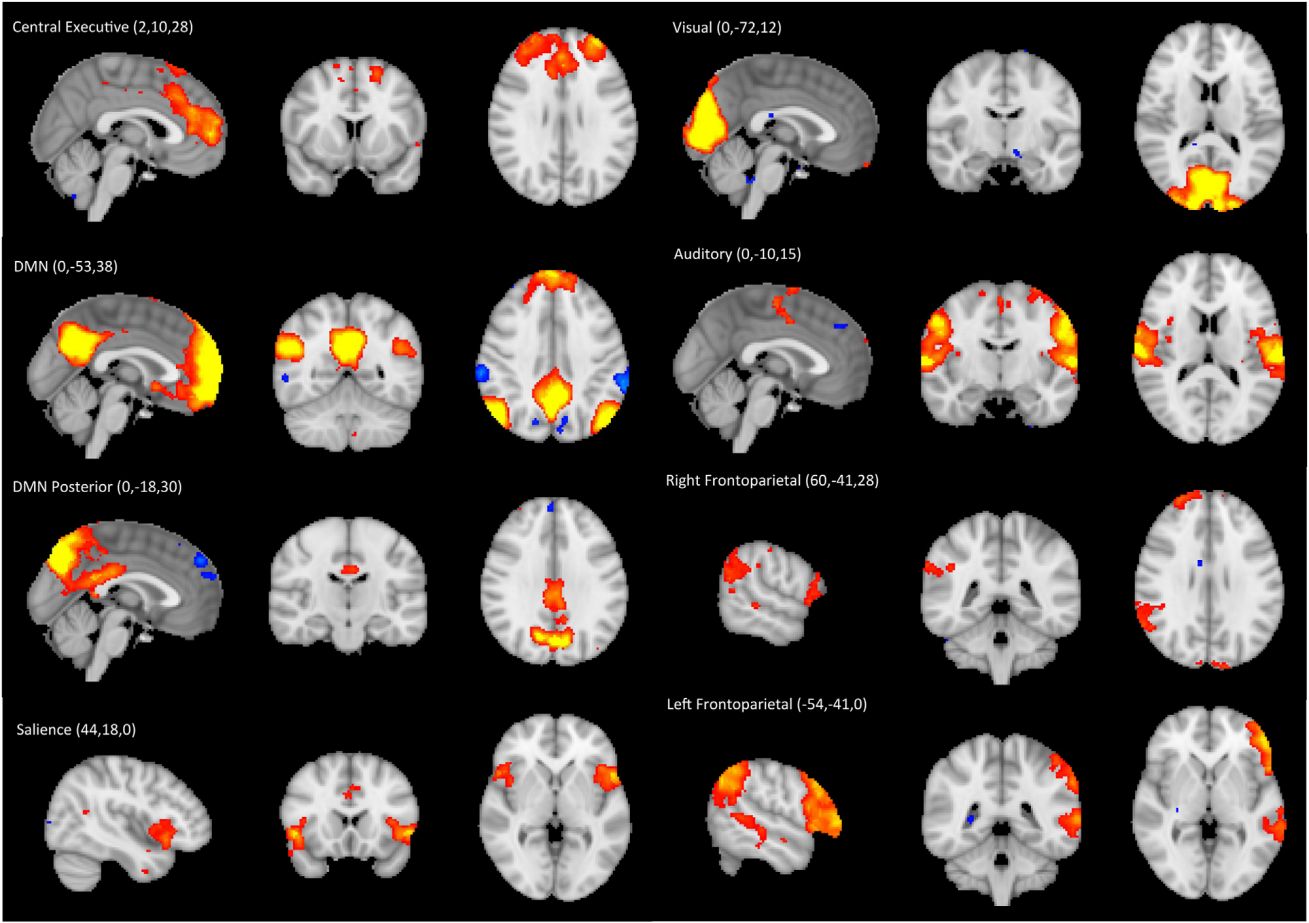
Resting-state networks identified in the study. Resting-state networks are shown in axial, sagittal, and coronal views overlaid onto the standard Montreal Neurological Institute brain. All maps were thresholded at 3.1. Montreal Neurological Institute coordinates for each resting-state network location are detailed and marked with crosshairs. Red signifies a positive correlation and blue signifies a negative correlation. DMN, default mode network.

Out of the 20 independent components, 8 were clearly identified as RSNs, with the remainder being at least partially noise, due to physiological factors, head motion, and artifacts from the scanner. All identified RSNs are displayed in Figure 2.

**Figure 3:**
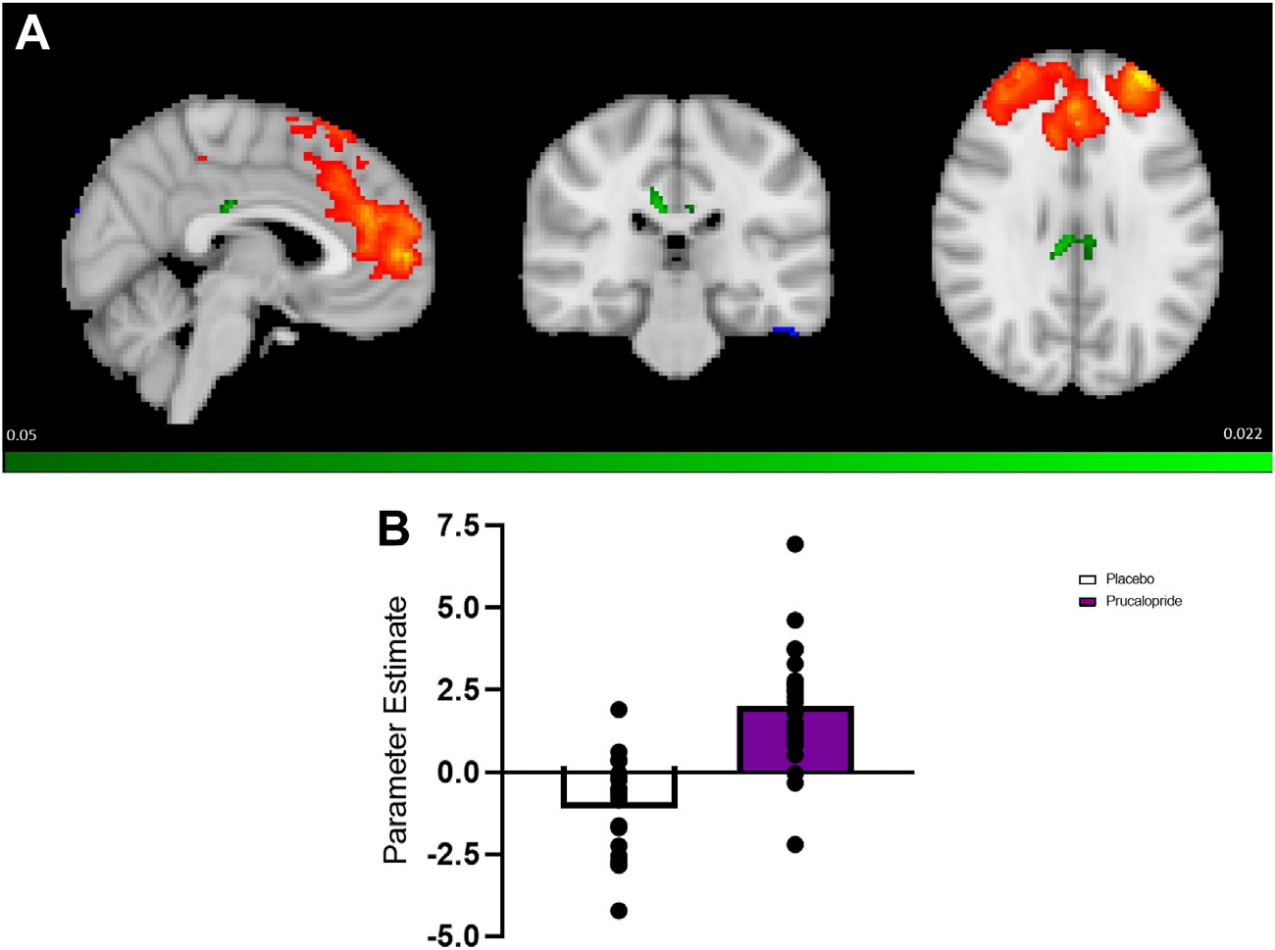
Network analysis showing greater resting-state functional connectivity between the posterior and anterior cingulate cortices cluster and central executive network. **(A)** Network maps. Maps show axial/sagittal/coronal slices of *z*-statistic images thresholded to 3.1. The central executive network is shown in red/blue. The significant posterior and anterior cingulate cortices cluster is shown in green (prucalopride > placebo; tmax = 4.8, *p =* .032; Montreal Neurological Institute peak voxel: x = 5, y = −28, z = 28; cluster size = 94 voxels; green bar indicates *p* [significance < .05], shown at Montreal Neurological Institute coordinates x = 3, y = −23, z = 28). **(B)** Graph showing the mean parameter estimate in the prucalopride group (purple) vs. placebo group (white) representing the change in resting-state functional connectivity in the whole-brain posterior and anterior cingulate cortices cluster relative to the central executive network. Data points are shown as a scatter plot.

The prucalopride group, when compared with the placebo group, showed significantly greater rsFC between the CEN and a cluster predominately involving the PCC extending into the ACC (prucalopride > placebo; tmax = 4.8, *p =* .032; MNI peak voxel: x = 8, y = −28, *z* = 28; cluster size = 94 voxels) (see Figure 3A, B). Post hoc analyses did not identify any group-level correlations between treatment and cognitive scores (placebo > prucalopride: *p =* .62; prucalopride > placebo: *p =* .90).

#### Seed Analysis

**Figure 4:**
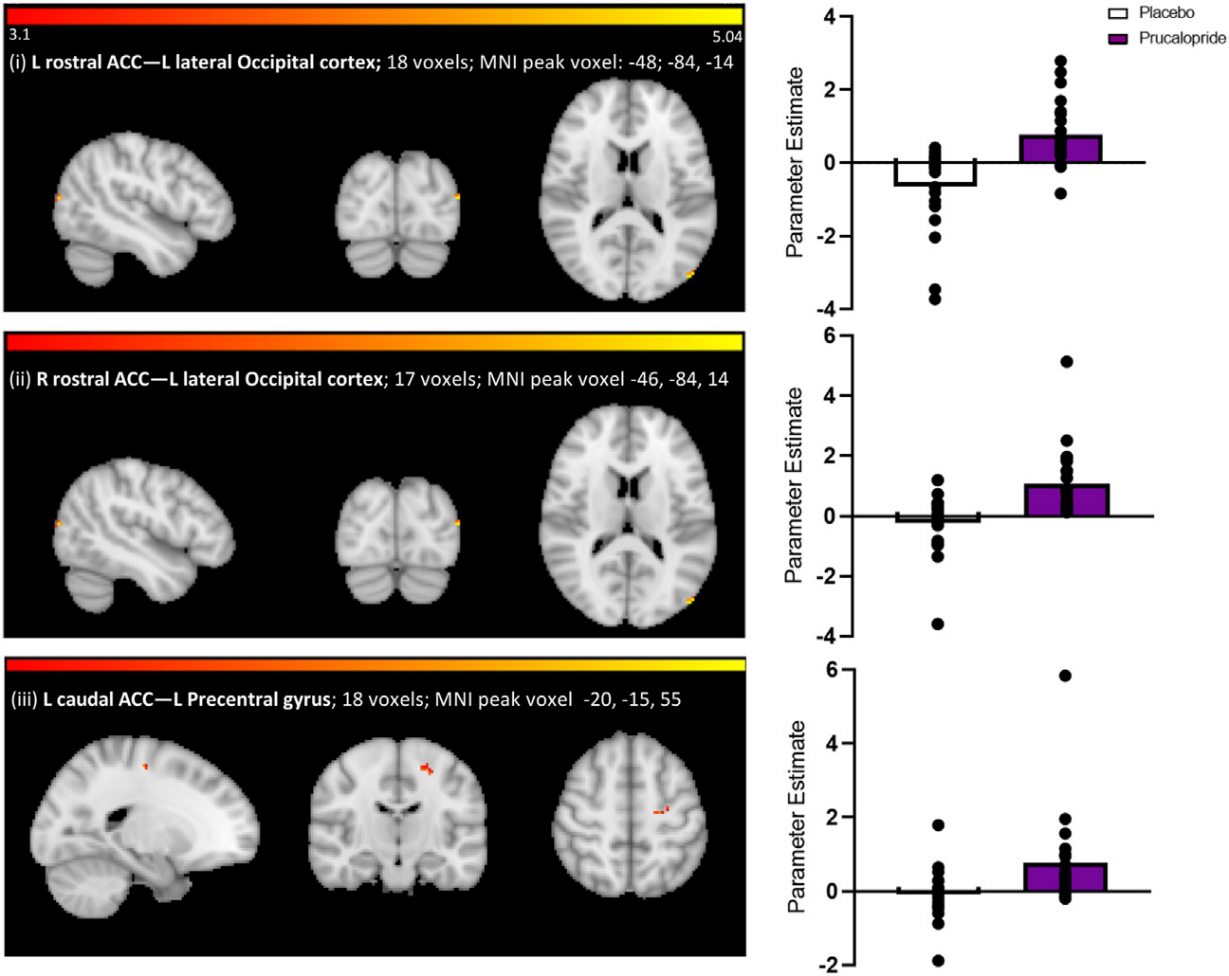
Network maps and corresponding parameter estimates for each seed result with significant resting-state functional connectivity changes with prucalopride: increased connectivity. Brain maps show axial/sagittal/coronal slices of *z*-statistic images thresholded to 3.1 with the cluster shown at the peak voxel location. The color bars indicate the *z* value. The size and Montreal Neurological Institute (MNI) peak voxels for clusters are shown within the figure. The *p* values shown were corrected to 3 decimal places. Parameter estimate of the resting-state functional connectivity cluster: placebo = white, prucalopride = purple, data points shown as a scatter plot. (i) Seed: left rostral anterior cingulate cortex (L rostral ACC); connectivity result: L lateral occipital cortex; prucalopride > placebo; *z =* 4.99, corrected *p =* .023. (ii) Seed: right rostral ACC (R rostral ACC); connectivity result: L lateral occipital cortex; prucalopride > placebo; *z =* 4.34, corrected *p =* .028. (iii) Seed: L caudal ACC; connectivity result: L precentral gyrus; prucalopride > placebo; *z =* 4.17, corrected *p =* .002.

**Figure 5:**
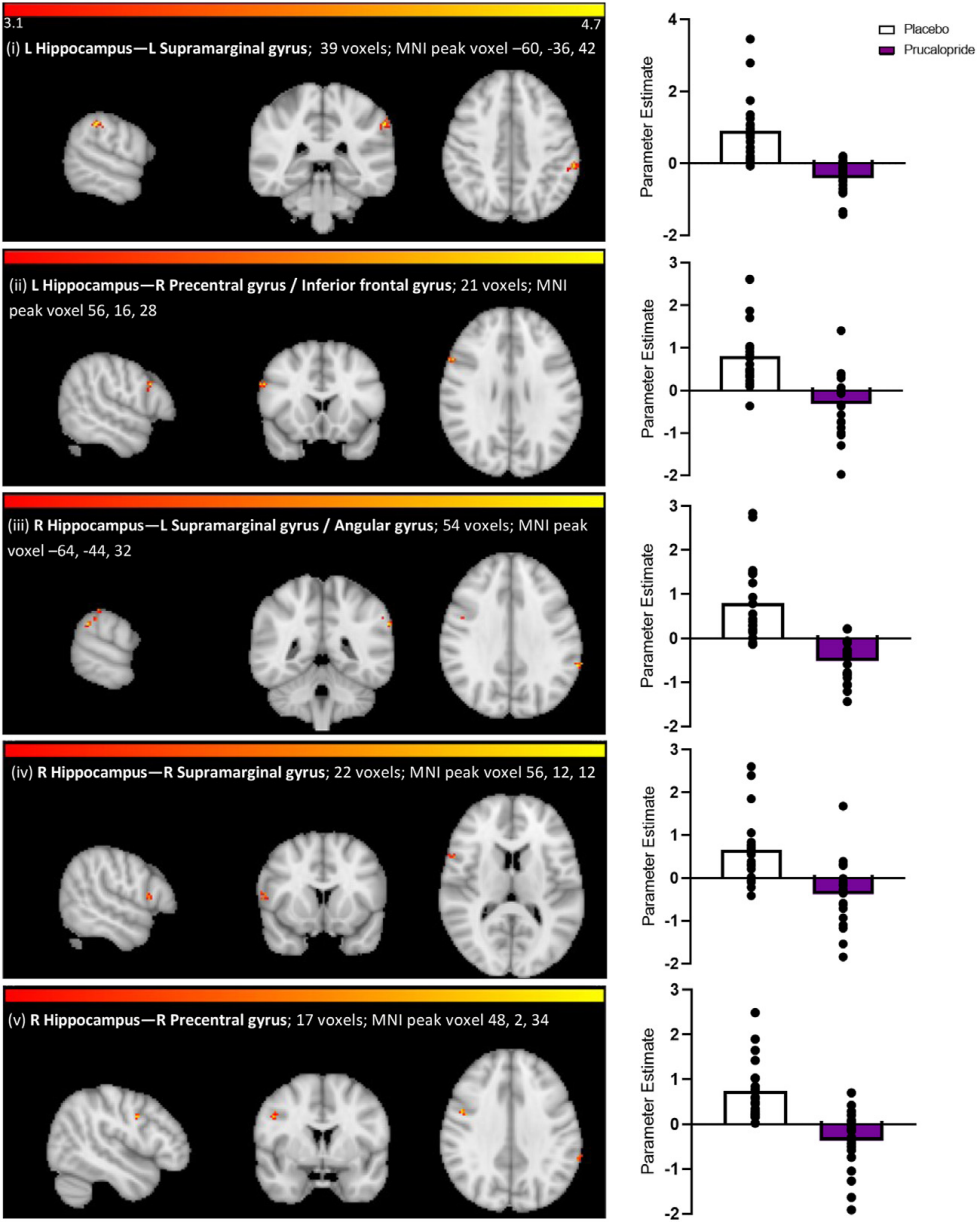
Network maps and corresponding parameter estimates for each seed result with significant resting-state functional connectivity changes with prucalopride: decreased connectivity. Brain maps show axial/sagittal/coronal slices of *z*-statistic images thresholded to 3.1 with the cluster shown at the peak voxel location. The color bars indicate the *z* value. The size and Montreal Neurological Institute (MNI) peak voxels for clusters are shown within figure. The *p* values shown were corrected to 3 decimal places. Parameter estimate of the resting-state functional connectivity cluster: placebo = white, prucalopride = purple, data points shown as a scatter plot. (i) Seed: left (L) hippocampus; connectivity result: L supramarginal gyrus; placebo > prucalopride; *z =* 4.65, corrected *p <* .001. (ii) Seed: L hippocampus; connectivity result: right (R) inferior frontal gyrus/precentral gyrus; placebo > prucalopride; *z =* 4.35, corrected *p =* .005. (iii) Seed: R hippocampus; connectivity result: L supramarginal gyrus/angular gyrus; placebo > prucalopride; *z =* 4.61, corrected *p* < .001. (iv) Seed: R hippocampus; connectivity result: R supramarginal gyrus; placebo > prucalopride; *z =* 3.89, corrected *p <* .004. (v) Seed: R hippocampus; connectivity result: R inferior frontal gyrus/precentral gyrus; placebo > prucalopride; *z =* 4.38, corrected *p =* .03.

Changes in connectivity between seed regions and other brain regions are summarized in Figures 4 and 5 and Table S2.

For the ACC, when compared with participants taking placebo, participants taking prucalopride showed greater connectivity between ACC subdivisions and other attentional regions: the left and right rostral/affective ACC and the left lateral occipital cortex (left rostral/affective ACC: prucalopride > placebo; *z =* 4.99, corrected *p <* .03; MNI peak voxel: x = −48, y = −84, *z* = 14; cluster size = 18 voxels; right rostral/affective ACC: prucalopride > placebo; *z =* 4.34, corrected *p <* .03; MNI peak voxel: *x* = −46, *y* = −84, *z* = 14; cluster size = 17 voxels) and the left caudal/cognitive ACC and the left precentral gyrus (left caudal/cognitive ACC: prucalopride > placebo; *z =* 4.17, corrected *p <* .002; MNI peak voxel: x = −20, y = −18, z = 56; cluster size = 18 voxels) (see Figure 4 and the Supplement). The right caudal/cognitive ACC was not significantly related to another region.

When compared with the placebo group, the prucalopride group also showed reduced connectivity between the left and right hippocampus and 1) the supramarginal gyrus/angular gyrus and 2) the precentral gyrus/inferior frontal gyrus, a set of regions analogous to the DMN (see Figure 5 and the Supplement). This pattern of reduced connectivity within the DMN was supported by additional findings of reduced connectivity involving seeds placed in the angular gyrus and PCC and their connections (to other regions in the DMN and visuo-language processing regions). However, although these survived correction for multiple comparisons at the voxel level, they did not survive correction for multiple comparisons at the seed level (see Table S2). Post hoc analyses did not identify any group-level correlations between treatment and cognitive scores (*p*s > .06).

#### Sensitivity Analyses

Results were similar when gray matter, perfusion maps, and sex were included as voxel-dependent explanatory variables of no interest for whole network and seed analyses (see Table S2 and Figure S2). We have previously shown that native language and cognitive task performance were not correlated (41,50), and native language was not correlated here with the CEN-PCC/ACC cluster parameter estimate (*p* > .9).

## Discussion

In healthy volunteers at rest, we found that 6 days of prucalopride administration was associated with greater rsFC between the PCC/ACC and the CEN. Additional seed analysis also identified 2 important findings in the prucalopride group compared with the placebo group: 1) greater rsFC between both the left and right rostral ACC and a visual attentional region, the lateral occipital cortex; and 2) reduced rsFC, as expected, within the DMN. In seed analyses, the precentral gyrus showed greater rsFC with the caudal ACC and reduced rsFC with DMN regions.

In support of our hypothesis, seed analysis demonstrated that prucalopride was associated with reduced rsFC between regions of the DMN, including the hippocampus, inferior parietal lobule (angular gyrus, supramarginal gyrus), and inferior frontal gyrus. The DMN is a set of regions that classically show intrinsic activity at rest and relatively reduced activity (i.e., deactivate) when focus is required for external cognitive tasks and internal demands (such as autobiographical memory retrieval) (60). A failure of the DMN to deactivate appropriately when required is associated with attentional deficits (61) and reduced cognitive performance in health and disease (49,62). Thus, the finding of reduced DMN rsFC is consistent with our previous work showing that acute and subacute prucalopride improves task-related cognition (40,41,50) and reduces activity in regions of the DMN during memory performance (50).

Our finding that 6 days of prucalopride administration was associated with greater rsFC between the CEN and the PCC/ACC builds on existing knowledge of the role of these regions in cognition. The PCC and ACC are both important regions in information processing and the regulation of information within the brain; the ACC has a variety of cognitive, affective, and sensorimotor roles and connections (51,52), whereas the PCC is known for its roles in internally directed cognition, arousal, and attention (63). The PCC (Brodmann areas 23 and 31) (64) seems to be particularly active during rest and when participants retrieve autobiographical memories, plan for the future, and decide where to focus attention (63). In particular, the PCC and ACC are both strongly implicated in the triple network model (45), i.e., they are both connected to the CEN and SN, and are key hubs within the DMN. Compared with healthy volunteers, those with remitted and current depression show functional changes and altered reciprocal connectivity within and between the individual networks of the triple network model (42, 43, 44,65,66). The function of these networks in relation to each other is thought to be important; dysfunction in one network may affect the others in the model, and appropriate integration of information across the model is required for optimal cognitive function (46).

The ACC also has an important role in attention, potentially mediating the effects of emotional interference, with the rostral ACC assessing the relevant information involved and the caudal ACC undertaking the control aspect (52,67) as a node of the SN (51). This is consistent with the findings of our study, in which we found greater connectivity in participants who had received prucalopride between the rostral ACC and lateral occipital cortex (a region that aids attention for objects that matter) (68) and between the caudal ACC and the precentral gyrus (a sensorimotor region that has been implicated in visuospatial attention, especially for unpredictable situations) (69). These findings also appear to be a reproduction of those by Esposito *et al.* (70), who also identified increased ACC–occipital cortex rsFC with the cognitive-enhancing drug modafinil.

An interesting facet of our results is that prucalopride was associated with both increased rsFC between regions of cognition networks and reduced rsFC within regions of the DMN. Existing literature supports this, despite some understandable variability depending on the agent involved and the population studied. Focusing on findings in healthy adults, Esposito *et al.* (70) found a similar pattern of change following an acute dose of 100 mg modafinil. Modafinil increased rsFC between the ACC and the CEN and between the lateral occipital cortex and dorsal attention network, as well as improving scores on a progressive matrices test (70). Another example using various procognitive medications found an increased rsFC between cognitive networks but reduced rsFC between the PCC and the DMN. Further analysis revealed that greater reductions in DMN rsFC were associated with better performance (47). Mueller *et al.* (71) suggested that methylphenidate both increases and decreases rsFC between cognitive and sensorimotor networks, including the ACC, PCC, precentral gyrus, and occipital cortices, all regions where rsFC was affected by prucalopride in the current study. These results are similar, although with some differences, to the effects of procognitive treatments within clinical populations [alcohol dependence (72), Alzheimer’s disease (73), stroke (74)]. We are not aware of studies examining the effect of procognitive medication on rsFC in depressed patients with cognitive impairment.

In summary, consistent with our previous (task-based) and current (rsFC) fMRI results with prucalopride, and consistent with findings with other procognitive agents, medications with cognitive-enhancing properties appear to produce 1) increased connectivity within cognitive networks (such as the CEN) and 2) reduced connectivity within the DMN and its connections (63,75). Moreover, it appears that a greater negative correlation between these two systems (cognitive networks vs. the DMN) may be associated with better behavioral performance (75). In this way, successful performance of cognitive demands appears dependent on the ability to dynamically modulate the DMN as well as the other cognitive networks within the triple network model; furthermore, effects within this network may be an important means by which procognitive medications act. However, DMN activity may also be responsible for synchronizing higher-order networks and helping the brain transition from one state to another (51,76). Thus, procognitive medications must ensure that DMN suppression is nuanced to ensure that effects are functionally advantageous.

Our results are consistent with the PCC and the ACC playing a key role in cognitive enhancement and perhaps acting as a pivot between the dual functions of these areas in attention and cognition (via the DMN and CEN in particular). That is, the PCC and ACC may help to control the balance between internally directed attention and externally directed focus (63), explaining why administration of a procognitive agent may both simultaneously increase rsFC within cognitive networks (as per our network analyses) and reduce task-based activation [as per (50)] and rsFC within regions of the DMN (as per our seed analyses).

Subdividing the ACC along anatomical lines as a proxy of function allowed us to take account of some of the complexity of the ACC in its actions within RSNs with the use of a procognitive agent. However, the transitional area between affective and cognitive functions is likely to be more complicated and gradual than the sharp distinctions necessary for analytic processes, and may be subject to interindividual variation (51). Furthermore, as this was a resting-state fMRI scan, we have assumed that the changes in rsFC seen here involving the hippocampus and angular gyrus relate to their role as part of the DMN, as opposed to their memory-processing functions, which is supported by other DMN regions interacting in a similar pattern in our study.

In terms of limitations, the results described here include some small clusters, although there is evidence of consistency within these. For example, the left and right rostral ACC showed increased connectivity with prucalopride for the same region (i.e., the left lateral occipital cortex). In this study, we used a low dose of prucalopride (1 mg, as opposed to the licensed dose of 2 mg) to reduce the risk of side effects. At this time, we currently lack information about the optimal dose of prucalopride needed for occupancy of brain 5-HT_4_ receptors, although data from our previous reports indicate that 1 mg is sufficient to demonstrate procognitive potential. The sample size required (17 per group to give 90% power with α = 5%) was calculated based on a conservative estimated effect size of 0.5 to 0.7 with behavioral analyses. However, in view of the less clear power calculations for fMRI data, we oversampled in each group. Unfortunately, power may have been affected due to preanalysis (*n =* 3) and intra-analysis (*n =* 3) exclusions. By chance, most participants whose first language was not English received placebo, but there was no verbal task included here, and network analysis parameter estimates did not correlate with native language, similar to findings with previous behavioral data.

We also did not perform baseline fMRI testing in this study due to learning effects that would result in the fMRI tasks being performed earlier in the sequence. Including behavioral scores from a hippocampal memory encoding task (undertaken with the same participants earlier in the MRI session) as a covariate of interest did not identify group-level correlations for either network or seed analysis (although here there was a trend for the left hippocampus seed after correction for multiple comparisons [correlation for placebo group with left lateral occipital cortex: *p =* .06]). However, these post hoc findings should be interpreted with caution, as this additional analysis was underpowered. Future studies designed to assess such brain–behavior correlations require large datasets (77). We also note that our findings may not generalize to other 5-HT_4_ receptor agonists.

In conclusion, the results from this analysis of resting-state fMRI data following 6 days of 1 mg prucalopride support our previous translational work and further suggest that prucalopride may affect cognition via effects on attentional neural networks: reducing connectivity within the DMN and between the DMN and its connections while also enhancing connectivity within networks involved in cognition.
