# Supplementary material for: 5-HT4 Receptor Agonist Effects on Functional Connectivity in the Human Brain: Implications for Procognitive Action
Source: Biol Psychiatry Cogn Neurosci Neuroimaging. 2023 Nov;8(11):1124–34. doi: 10.1016/j.bpsc.2023.03.014 (PMC10914664; doi:10.1016/j.bpsc.2023.03.014)
Supplement: Supplementary Material [file mmc1.pdf]

SUPPLEMENTARY INFORMATION

$5\text{-}HT_4$ Receptor Agonist Effects on Functional Connectivity in the Human Brain: Implications for Procognitive Action

de Cates *et al.*

Supplementary Material

Full details of questionnaire measures used

Participants completed self-report questionnaires to obtain baseline measures of mood, anxiety, and personality: Beck Depression Inventory-II, Snaith–Hamilton Pleasure Scale (SHAPS), Spielberger State-Trait Anxiety Inventory, Trait Version (STAI-T), and Eysenck Personality Questionnaire. In addition, affect and anxiety were measured longitudinally at baseline and two other times (baseline, preimaging (day 6), and post imaging (day 6)): affect using the Positive and Negative Affect Scale (PANAS), and the visual analogue scale (VAS), and for anxiety the Spielberger State-Trait Anxiety Inventory, State Version (STAI-S). Side effects were measured at these same three time points using a scale where participants rated the extent to which they were experiencing each of the most commonly reported side effects of prucalopride. At the end of the study (day 7), participants guessed their drug allocation with a forced-choice question.

Full details of fMRI methods

All testing took place at the Department of Psychiatry and the Oxford Centre for Human Brain Activity (OHBA), part of the Wellcome Integrative Neuroimaging Centre (WIN).

Pre-processing:

T1-weighted structural MRI and multi-echo resting state fMRI images were pre-processed using fMRIprep 20.2.0 (1) and a denoised optimially-combined timeseries file for each participant produced with Tedana 0.0.9 (2, 3, 4, 5). Group-level network analysis was conducted with the FMRIB toolbox. Full preprocessing details can be found in Supplementary Material, but the outline is explained briefly here. Pre-processing code is available at (6).

Resting-state fMRI data were unwarped using acquired B0 field maps, co-registered to the reference run and the T1w reference using boundary-based registration. Following this, resting-state fMRI data were registered to standard space using linear and non-linear tools. Principal components were estimated after high-pass filtering the preprocessed BOLD time-series (using a discrete cosine filter with 128s cut-off).

Tedana version 0.0.9 (5) produced a denoised optimally-combined time-series file for each participant, using input data from fMRIprep. A mask using the first echo for each participant was applied to the data, and then multi-echo data were optimally combined using the T2* combination method (7).

fMRIprep: Results included in this manuscript come from preprocessing performed using fMRIPrep 20.2.0 (1) , which is based on Nipype 1.5.1 (8, 9).

Anatomical data preprocessing: The T1-weighted (T1w) image was corrected for intensity non-uniformity (INU) with N4BiasFieldCorrection (10), and used as T1w-reference throughout the workflow. The T1w-reference was then skull-stripped with a Nipype implementation of the antsBrainExtraction.sh workflow (from ANTs), using OASIS30ANTs as target template. Brain tissue segmentation of cerebrospinal fluid (CSF), white-matter (WM) and gray-matter (GM) was performed on the brain-extracted T1w using fast (FSL 5.0.9, RRID:SCR_002823, (11). Volume-based spatial normalization to two standard spaces (MNI152NLin6Asym, MNI152NLin2009cAsym) was performed through nonlinear registration with antsRegistration (ANTs 2.3.3), using brain-extracted versions of both T1w reference and the T1w template. Head-motion parameters with respect to the BOLD reference (transformation matrices, and six corresponding rotation and translation parameters) were estimated using MCFLIRT (12).

Functional data preprocessing: For each of the 1 BOLD runs found per subject, a reference volume and its skull-stripped version were generated by aligning and averaging the first echo of 3 single-band references (SBRefs). Head-motion parameters with respect to the BOLD reference (transformation matrices, and six corresponding rotation and translation parameters) are estimated before any spatiotemporal filtering using mcflirt (FSL 5.0.9, (12). A B0-nonuniformity map (or fieldmap) was estimated based on a phase-difference map calculated with a dual-echo GRE (gradient-recall echo) sequence, processed with a custom workflow of SDCFlows (13). The fieldmap was then co-registered to the target EPI (echo-planar imaging) reference run and converted to a displacements field map with FSL\u2019s fugue and other SDCflows tools. Based on the estimated susceptibility distortion, a corrected EPI (echo-planar imaging) reference was calculated for a more accurate co-registration with the anatomical reference. The BOLD reference was then co-registered to the T1w reference using flirt (FSL 5.0.9, (14)) with the boundary-based registration (15) cost-function. Co-registration was configured with nine degrees of freedom to account for distortions remaining in the BOLD reference. A T2* map was estimated from the preprocessed BOLD by fitting to a monoexponential signal decay model with nonlinear regression, using T2*/S0 estimates from a log-linear regression fit as initial values. For each voxel, the maximal number of echoes with reliable signal in that voxel were used to fit the model. The calculated T2* map was then used to optimally combine preprocessed BOLD across echoes following the method described in (7). The optimally combined time series was carried forward as the preprocessed BOLD. The BOLD time-series were resampled into standard space, generating a

preprocessed BOLD run in MNI152NLin6Asym space. Several confounding time-series were calculated based on the preprocessed BOLD: framewise displacement (FD), DVARS and three region-wise global signals. The three global signals are extracted within the CSF, the WM, and the whole-brain masks. Additionally, a set of physiological regressors were extracted to allow for component-based noise correction (CompCor, (16). Principal components are estimated after high-pass filtering the preprocessed BOLD time-series (using a discrete cosine filter with 128s cut-off) for the two CompCor variants: temporal (tCompCor) and anatomical (aCompCor). The head-motion estimates calculated in the correction step were also placed within the corresponding confounds file. The confound time series derived from head motion estimates and global signals were expanded with the inclusion of temporal derivatives and quadratic terms for each (17). Frames that exceeded a threshold of 0.5 mm FD or 1.5 standardised DVARS were annotated as motion outliers. All resamplings can be performed with a single interpolation step by composing all the pertinent transformations (i.e. head-motion transform matrices, susceptibility distortion correction when available, and co-registrations to anatomical and output spaces). Gridded (volumetric) resamplings were performed using antsApplyTransforms (ANTs), configured with Lanczos interpolation to minimize the smoothing effects of other kernels (18). Non-gridded (surface) resamplings were performed using mri_vol2surf (FreeSurfer).

Tedana: Tedana version 0.0.9 was used to produce a denoised optimally combined timeseries file for each participant. TE-dependence analysis was performed on input data from fMRIprep. A user-defined mask was applied to the data. An adaptive mask was then generated, in which each voxel's value reflects the number of echoes with 'good' data. A monoexponential model was fit to the data at each voxel using log-linear regression in order to estimate T2* and S0 maps. For each voxel, the value from the adaptive mask was used to determine which echoes would be used to estimate T2* and S0. Multi-echo data were then optimally combined using the T2* combination method (7). Principal component analysis based on the PCA component estimation with a Moving Average (stationary Gaussian) process (19) was applied to the optimally combined data for dimensionality reduction. A series of TE-dependence metrics were calculated for each component, including Kappa, Rho, and variance explained. Independent component analysis was then used to decompose the dimensionally reduced dataset. Next, component selection was performed to identify BOLD (TE-dependent), non-BOLD (TE-independent), and uncertain (low-variance) components using the Kundu decision tree (v2.5; (2)). This workflow used numpy (20), scipy (21), pandas (22), scikit-learn (23), nilearn, and nibabel (24). This workflow also used the Dice similarity index (25).

The pre-processed cleaned functional data were temporally concatenated across subjects and decomposed into ICs using FSL MELODIC. Dimensionality estimation for group maps was set to 20 IC maps, which is an approximate average of the individual IC maps thresholds as determined by spatial mixture modelling. They were identified as either belonging to the most frequently reported major RSNs (26), or reflecting movement, physiological or scanner noise. This was done by visual inspection, independently by AdeC and MM, and additionally through a comparison to previously published maps (26) using Pearson spatial cross-correlation. Next, the set of spatial maps from the group-average analysis was used to generate subject-specific versions of the spatial maps, and associated timeseries, using dual regression (27, 28). First, for each subject, the group-average set of spatial maps was

regressed (as spatial regressors in a multiple regression) into the subject's 4D space–time dataset. This resulted in a set of subject-specific timeseries, one per group-level spatial map. Next, those timeseries were regressed (as temporal regressors, again in a multiple regression) into the same 4D dataset, resulting in a set of subject-specific spatial maps, one per group-level spatial map. We then tested for statistically significant differences between the groups using FSL's randomize permutation-testing tool (5000 permutations). Cluster-based thresholding was applied using Threshold-Free-Cluster-Enhancement (TFCE) approach and a family-wise-error corrected cluster significance threshold of $p < 0.05$ applied to the suprathreshold clusters (29).

Seed analysis: For the seed analysis, pre-determined region-specific masks were chosen: anterior cingulate cortex (ACC), posterior cingulate cortex (PCC), hippocampus, amygdala, angular gyrus. As the ACC is a large structure with multiple roles (often summarised as affective versus cognitive), this region was subdivided into different seeds to reflect this distinction along functional lines (Vogt, Uddin) as per the FSL Talairach atlas: a rostral "affective" ACC (Brodmann areas rostral 24(a), 25, 33, rostral 32(a)) and a caudal "cognitive" ACC (Brodmann areas caudal 24(b), caudal 32(b+c)). Masks were binarized and thresholded at 50% before creating a standard to high resolution matrix, which was applied to each mask for each participant in turn to register the mask into each individual's functional (EPI) space. We then extracted the time series for each mask for each participant, as well as a white matter and CSF mask. We used FSL FEAT to perform group-level analysis with two explanatory variables (prucalopride versus placebo) testing for contrasts placebo > prucalopride and prucalopride > placebo. Cluster-based thresholding (Z>3.1) was used to identify significant clusters for each seed analysis. To harmonise this process with FSL, we used files created with our data by FSL's MELODIC ICA as well as Tedana.

***Oxford_ASL*:** This performs label-control subtraction, inference of voxel-wise perfusion, and voxel-wise calibration to obtain absolute perfusion maps, and controls for partial volume effects at the single-subject level (30, 31). These were non-linearly aligned with standard space via an initial linear transformation T1 structural space, followed by the application of the non-linear warp. Normalised images were merged and smoothed with a Gaussian smoothing kernel of 2.35 mm (to match resting state data) to enable use as a voxel-dependent explanatory variable of no interest in the analysis.

Supplementary Tables:

Supplementary Table 1: Pearson spatial cross-correlation (PCC) quantitative comparison of Resting State Networks (RSNs) found in the study to previously published maps (Smith et al., 2009).

**Canonical RSN**	**Agreement with Smith (PCC)**
Visual	0.7
DMN	0.6
DMN (Posterior)	0.4
Sensorimotor	0.4
Auditory	0.4
Executive control (CEN)	0.6
Right Frontoparietal	0.3
Left Frontoparietal	0.6

Supplementary Table 2: Details of seed results with significant resting state functional connectivity (rsFC) changes with prucalopride (significant corrected values *p* values in BOLD)

Seed region	Connectivity result	Cope	Z score	P value	Corrected P value (Bonferroni)	MNI peak voxel (x)	(y)	(z)	Cluster size (voxels)	Cluster size (voxels) adjustment for (i) GM (ii) ASL+GM (iii) sex
L rostral ACC	L lateral Occipital cortex	Prucalopride > placebo	4.99	0.00379	**0.02274**	-48	-84	14	18	(i) 15<br>(ii) 19<br>(iii) 18
R rostral ACC	L lateral Occipital cortex	Prucalopride > placebo	4.34	0.00482	**0.02892**	-46	-84	14	17	(i) 18<br>(ii) 18<br>(iii) 17
L caudal ACC	L Precentral gyrus	Prucalopride > placebo	4.17	0.00323	**0.00194**	-20	-18	56	18	(i) 18<br>(ii) 13<br>(iii) 14
L Hippocampus	L Supramarginal gyrus	Placebo > prucalopride	4.65	<0.000001	**<0.00001**	-60	-36	42	39	(i) 39<br>(ii) 43<br>(iii) 37
	R Inferior frontal gyrus / Precentral gyrus	Placebo > prucalopride	4.35	0.000765	**0.00459**	56	16	28	21	i) 22<br>ii) 25<br>iii) 20
R Hippocampus	L Supramarginal gyrus / Angular gyrus	Placebo > prucalopride	4.61	<0.000001	**<0.00001**	-64	-32	44	54	(i) 48<br>(ii) 51<br>(iii) 52

	R Supramarginal gyrus	Placebo > prucalopride	3.89	0.000668	**0.00401**	56	12	12	22	(i) 14<br>(ii) 13<br>(iii) 23
	R Inferior frontal gyrus / Precentral gyrus	Placebo > prucalopride	4.38	0.00532	**0.03192**	48	2	34	17	(i) 16<br>(ii) 16<br>(iii) 18
Angular gyrus	L Inferior temporal gyrus / Occipital fusiform cortex	Placebo > prucalopride	4.23	0.0178	0.1068	50	-54	-22	15	(i) 20<br>(ii) 13<br>(iii) 15
	L Inferior frontal gyrus / Precentral gyrus	Placebo > prucalopride	3.6	0.0275	0.165	-50	10	16	14	(i) 23<br>(ii) 22<br>(iii) Not survive voxel correction
PCC	L Supramarginal gyrus	Placebo > prucalopride	3.75	0.0103	0.0618	-60	-32	42	16	(i) 14<br>(ii) 18<br>(iii) 14

<u>Supplementary Figure 1</u>

Flow of participants for resting state fMRI scan of Seven-day prucalopride study

CONSORT
TRANSPARENT REPORTING of TRIALS

CONSORT 2010 Flow Diagram

Seven-day prucalopride – fMRI resting state

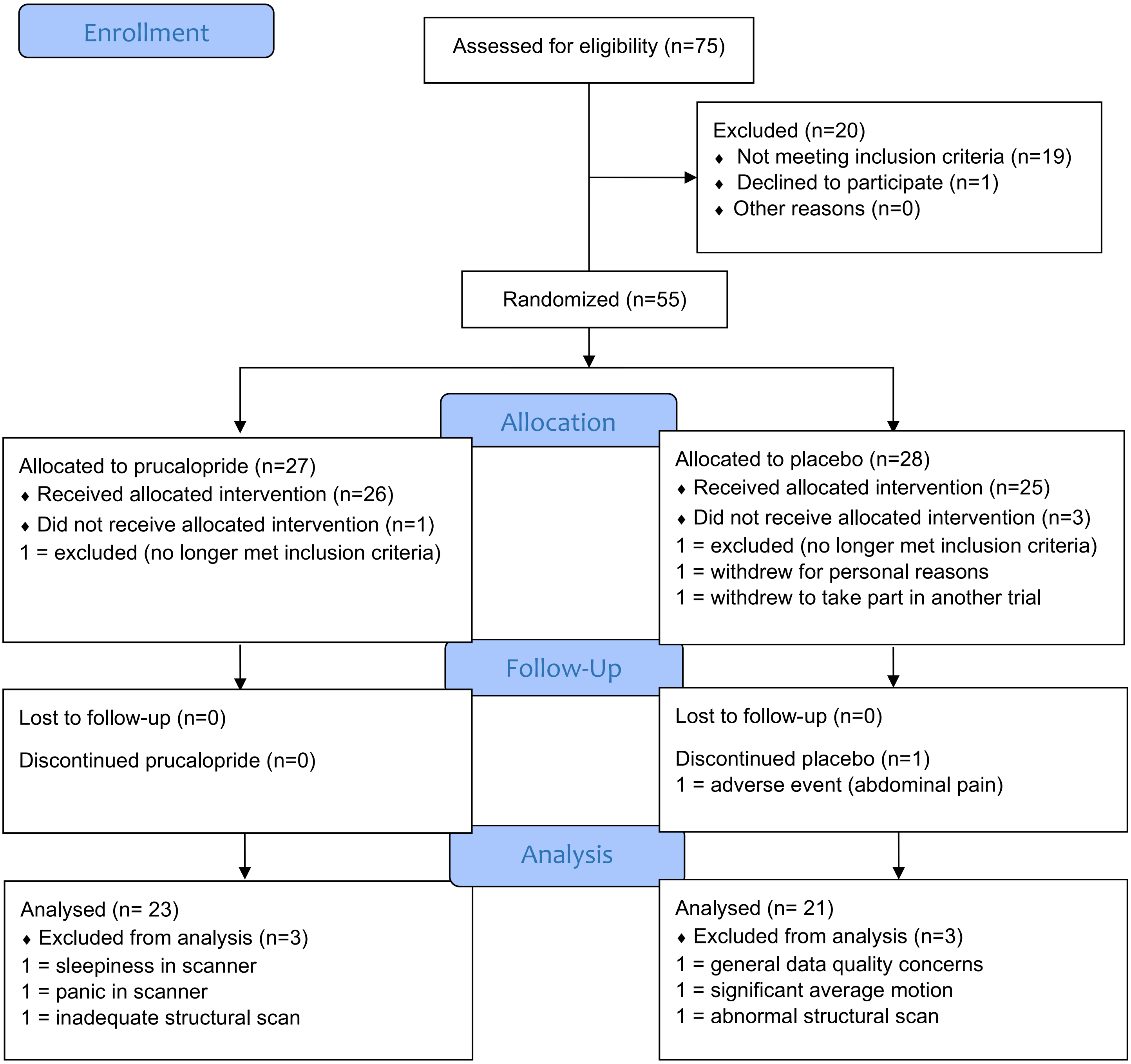

Supplementary Figure 2

Network maps with sex as a covariate of no interest. CEN shown in red/blue. Significant PCC / ACC cluster in green (prucalopride > placebo, tmax=4.2, p=0.037, MNI peak voxel x=10, y=-28, z=30, cluster size=45 voxels, colour bar indicates *p* (significance <0.05), shown at MNI co-ordinates 3.-23.28

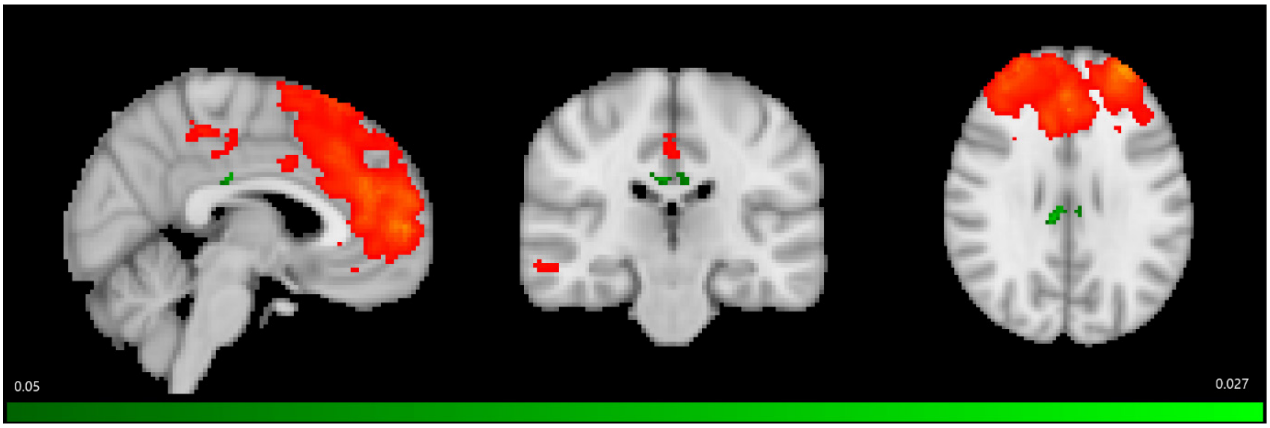

Supplementary References

1. Esteban O, Markiewicz CJ, Blair RW, Moodie CA, Isik AI, Erramuzpe A, *et al*. 2019. fMRIPrep: a robust preprocessing pipeline for functional MRI. *Nat Methods*. 16(1):111-6.
2. Kundu P, Brenowitz ND, Voon V, Worbe Y, Vértes PE, Inati SJ, *et al*. 2013. Integrated strategy for improving functional connectivity mapping using multiecho fMRI. *Proc Natl Acad Sci U S A*. 110(40):16187-92.
3. Kundu P, Inati SJ, Evans JW, Luh WM, Bandettini PA. 2012. Differentiating BOLD and non-BOLD signals in fMRI time series using multi-echo EPI. *Neuroimage*. 60(3):1759-70.
4. DuPre EM, Salo T, Ahmed Z, Bandettini PA, Bottenhorn KL, Caballero-Gaudes C, *et al*. 2021. TE-dependent analysis of multi-echo fMRI with tedana. Journal of Open Source Software. 6(66):3669.
5. The tedana Community, Zaki A, Bandettini PA, Bottenhorn KL, Caballero-Gaudes C, Dowdle LT, *et al*. 2022. ME-ICA/tedana: 0.0.12. *Zenodo*.
6. de Cates AN, Martens MAG, Wright LC, Gould van Praag CD, Capitao LP, Gibson D, *et al*. 2022. Analysis code for "Seven Day Prucalopride". *Zenodo*.
7. Posse S, Wiese S, Gembris D, Mathiak K, Kessler C, Grosse-Ruyken ML, *et al*. 1999. Enhancement of BOLD-contrast sensitivity by single-shot multi-echo functional MR imaging. *Magn Reson Med*. 42(1):87-97.
8. Gorgolewski K, Burns CD, Madison C, Clark D, Halchenko YO, Waskom ML, *et al*. 2011. Nipype: a flexible, lightweight and extensible neuroimaging data processing framework in python. *Front Neuroinform*. 5:13.
9. Gorgolewski KJ, Nichols T, Kennedy DN, Poline JB, Poldrack RA. 2018. Making replication prestigious. *Behav Brain Sci*. 41:e131.
10. Tustison NJ, Avants BB, Cook PA, Zheng Y, Egan A, Yushkevich PA, *et al*. 2010. N4ITK: improved N3 bias correction. *IEEE Trans Med Imaging*. 29(6):1310-20.
11. Zhang Y, Brady M, Smith S. 2001. Segmentation of brain MR images through a hidden Markov random field model and the expectation-maximization algorithm. *IEEE Trans Med Imaging*. 20(1):45-57.
12. Jenkinson M, Bannister P, Brady M, Smith S. 2002. Improved optimization for the robust and accurate linear registration and motion correction of brain images. *Neuroimage*. 17(2):825-41.
13. Glasser MF, Sotiropoulos SN, Wilson JA, Coalson TS, Fischl B, Andersson JL, *et al*. 2013. The minimal preprocessing pipelines for the Human Connectome Project. *Neuroimage*. 80:105-24.
14. Jenkinson M, Smith S. 2001. A global optimisation method for robust affine registration of brain images. *Med Image Anal*. 5(2):143-56.
15. Greve DN, Fischl B. 2009. Accurate and robust brain image alignment using boundary-based registration. *Neuroimage*. 48(1):63-72.
16. Behzadi Y, Restom K, Liau J, Liu TT. 2007. A component based noise correction method (CompCor) for BOLD and perfusion based fMRI. *Neuroimage*. 37(1):90-101.
17. Satterthwaite TD, Elliott MA, Gerraty RT, Ruparel K, Loughead J, Calkins ME, *et al*. 2013. An improved framework for confound regression and filtering for control of motion artifact in the preprocessing of resting-state functional connectivity data. *Neuroimage*. 64:240-56.
18. Lanczos C. 1964. A Precision Approximation of the Gamma Function. *Journal of the Society for Industrial and Applied Mathematics, Series B: Numerical Analysis*. 1:86-96.
19. Li YO, Adali T, Calhoun VD. 2007. Estimating the number of independent components for functional magnetic resonance imaging data. *Hum Brain Mapp*. 28(11):1251-66.
20. Van Der Walt S, Colbert S, Varoquaux G. 2011.The NumPy array: a structure for efficient numerical computation. *Computing in Science & Engineering*. 13:22.
21. Jones E, Oliphant E, Peterson P. 2001. SciPy: Open Source Scientific Tools for Python - [Available from: http://www.scipy.org/].

22. McKinney W. 2010. Data structures for statistical computing in python. *Proceedings of the 9th Python in Science Conference*. p. 51-6.
23. Pedregosa, F, Varoquaux G, Gramfort A, Michel V, Thirion B, *et al*. 2011. Scikit-learn: Machine learning in Python. *Journal of machine learning research*. 2825-30.
24. Brett M, Markiewicz C, Hanke M, Côté M-A, Cipollini B, McCarthy P, *et al*. nipy/nibabel: 2.4.1 (2.4.1). *Zenodo.*
25. Dice, LR. 1945. Measures of the amount of ecologic association between species. *Ecology*. 26(3):297-302.
26. Smith SM, Fox PT, Miller KL, Glahn DC, Fox PM, Mackay CE, *et al*. 2009. Correspondence of the brain's functional architecture during activation and rest. *Proc Natl Acad Sci U S A.* 106(31):13040-5.
27. Filippini N, MacIntosh BJ, Hough MG, Goodwin GM, Frisoni GB, Smith SM, *et al*. 2009. Distinct patterns of brain activity in young carriers of the APOE-epsilon4 allele. *Proc Natl Acad Sci U S A.* 106(17):7209-14.
28. Beckmann CF, DeLuca M, Devlin JT, Smith SM. 2005. Investigations into resting-state connectivity using independent component analysis. *Philos Trans R Soc Lond B Biol Sci.* 360(1457):1001-13.
29. Smith SM, Nichols TE. 2009. Threshold-free cluster enhancement: Addressing problems of smoothing, threshold dependence and localisation in cluster inference. *NeuroImage.* 44(1):83-98.
30. Chappell MA, Groves AR, MacIntosh BJ, Donahue MJ, Jezzard P, Woolrich MW. 2011. Partial volume correction of multiple inversion time arterial spin labeling MRI data. *Magn Reson Med.* 65(4):1173-83.
31. Chappell MA, Groves AR, Whitcher B, Woolrich MW. 2009. Variational Bayesian inference for a non-linear forward model. *IEEE Transactions on Signal Processing.* 57(1):223-36.
